# A Facile Synthesis Procedure for Sulfonated Aniline Oligomers with Distinct Microstructures

**DOI:** 10.3390/ma11091755

**Published:** 2018-09-18

**Authors:** Ramesh Karunagaran, Campbell Coghlan, Diana Tran, Tran Thanh Tung, Alexandre Burgun, Christian Doonan, Dusan Losic

**Affiliations:** 1 School of Chemical Engineering, University of Adelaide, Adelaide, SA 5005, Australia; ramesh.karunagaran@adelaide.edu.au (R.K.); Diana.tran@adelaide.edu.au (D.T.); tran.tung@adelaide.edu.au (T.T.T.); 2 School of Chemistry, University of Adelaide, Adelaide, SA 5005, Australia; cam.coghlan@adelaide.edu.au (C.C.); alexandre.burgun@adelaide.edu.au (A.B.); christian.doonan@adelaide.edu.au (C.D.)

**Keywords:** polyaniline, sulfonated polyaniline, microstructures, phenazine, pH

## Abstract

Well-defined sulfonated aniline oligomer (SAO) microstructures with rod and flake morphologies were successfully synthesized using an aniline and oxidant with a molar ratio of 10:1 in ethanol and acidic conditions (pH 4.8). The synthesized oligomers showed excellent dispersibility and assembled as well-defined structures in contrast to the shapeless aggregated material produced in a water medium. The synergistic effects among the monomer concentration, oxidant concentration, pH, and reaction medium are shown to be controlling parameters to generate SAO microstructures with distinct morphologies, whether micro sheets or micro rods.

## 1. Introduction

Polyaniline (PANI) emerged as the first conducting polymer whose electronic properties can be altered by protonation and charge-transfer doping [1,2]. Although PANI was initially synthesized in the 19th century, extensive research began after Epstein et al. [3] reported that the protonation of the emeraldine form of PANI could be transferred into a synthetic metal. Since this discovery, PANI is widely used for many applications including corrosion protection of metals [4], rechargeable batteries [5], super capacitors [6], organic field transistors [7], polymer light diodes [8], solar cells [9], and energy storage and conversion [2]. 

PANI is synthesized by the chemical oxidation process of aniline using a strong oxidizing agent such as ammonium persulfate (APS) in an acidic aqueous medium (e.g., 1 M hydrochloric acid (HCl)) [10]. The properties and the molecular structures of PANI can be controlled using different synthetic routes. These changes are associated with the monomer, oxidant, and dopant concentrations, nature of the dopant, molar ratio of aniline to oxidant and dopant, reaction temperature and medium, or pH [11,12]. In a typical PANI synthesis, both oligomeric and polymeric structures are formed, each releasing protons when the aniline molecules bind to each other, resulting in a decrease in solution pH [13]. The microstructures of PANI form a variety of structures including sheets, flakes, tubes, rods, tower-like rods, spheres, and granules [10,11]. Since aniline and its polymerization products are organic bases, the pH of the medium alters the surface chemistry and the reaction kinetics to enable its polymerization products to align as diverse structures [10].

Aniline is a weak base with a protonation constant (pKa) of 4.5, it exists as neutral and protonated aniline depending on the acidity of the reaction medium. According to Stejskal et al. [14], both neutral aniline and anilinium cations coexist equally at pH 4.6. At higher pH values, neutral aniline dominates in the reaction medium, and, under strong acidic conditions, anilinium cations excessively prevail. These two species have different oxidation potentials and oxidize at different rates. For example, neutral aniline has a lower oxidation potential than the anilinium cation [14]. A typical PANI synthesis using aniline and APS proceeds via three oxidation phases in a mildly acidic medium. Initially, the oxidation of the neutral aniline molecule occurs at pH > 3.5 with a fast and exothermic reaction to produce aniline oligomers. At a pH lower than 3.5, the material proceeds through an induction period of polymerization of aniline. Finally, at pH < 2.5, fast exothermic polymerization of anilinium cations occurs by releasing large number of protons to produce PANI. The highly conjugated conductive PANI synthesized at low pH is widely used in optical [15], catalytic, and electrical [16] applications. However, the multidimensional structures produced by PANI disperse poorly in water due to their large size, and obtaining microstructures with good dispersibility and processability remains a challenge [17].

To overcome its poor dispersibility required for many of these applications, Yue et al. [1] further modified the chemical structure of PANI by inserting a sulfonic group (SO_3_H) into PANI and reported the first self-doped high-molecular-weight sulfonic-acid ring-substituted conductive polyaniline in 1990. The SO_3_H groups in the PANI chain altered the properties of the parent material and provided unique properties such as self-doping, increased solubility in aqueous and organic solvents, environmental stability, thermal stability, and easy processability, as well as chemical, electrochemical, and optical properties that differed to those of PANI [18,19,20,21]. However, the structural properties of PANI or sulfonated PANI are yet to be fully investigated, because the synthesized products consist of larger and less ordered domains, entanglement of chains, polydispersity, and coils, and interacts with solvents in the reaction medium. Therefore, defined molecular structures such as oligomers with flexible side groups could provide a model for understanding molecular arrangements, providing attractive properties, and being tuned for use in different applications. 

The self-assembly of aniline oligomers and their structures were investigated and reported in the past [22,23,24,25]. Several investigations were carried out to identify the formation of aniline oligomers, and the reported evidence suggests that they are formed at pH > 3.5 where reactive neutral aniline is freely present [10,11,12,13,14,26,27]. At this pH, neutral aniline is easily oxidized to form *ortho*- or *para*-coupled aniline molecules via the reduction of APS, yielding phenazine-like structures [11]. Zujovic et al. [27] investigated the morphology of microstructures formed at higher pH and reported the formation of thin nanosheets formed by an inter chain ordering of oligomer products with lamellar structures via intermolecular hydrogen bonding (H-bonding) and π–π stacking interactions. These hydrophobic nanosheets have high surface area and stack to form layered sheets with lamellar structures, or curl as nanotubes to reduce their surface energy [13]. However, synthesis of these nanostructures becomes unfeasible because the polymerization reaction proceeds through a “falling pH” process to produce granular PANI structures at low pH values (pH < 2). As the reaction proceeds, the acidity of the medium increases following the release of two hydrogen atoms when the aniline polymer chain is formed, resulting in the formation of sulfuric acid [13]. Therefore, to obtain oligomeric nanostructures, the reaction should be conducted in a slightly acidic medium (pH > 3.5) without undergoing the “falling pH” effect. Huang et al. [12] synthesized well-dispersed PANI nanotubes in methanol, which suppressed the agglomeration of the nanostructures produced. The methanol molecules also formed strong intermolecular H-bonding with PANI [28] and pushed the polymer chains apart by forming hydrogen bonds perpendicular to the PANI chains [29]. The ethanol molecules allowed the PANI particles to be well-dispersed molecular structures with defined morphology [30]. Therefore, ethanol was used as the medium in the polymerization process to synthesize well-dispersed nanostructures with a defined morphology. 

In this paper, to address the limitation of previous procedures, a facile approach for synthesizing well-dispersed homogeneous sulfonated aniline oligomers with microstructures consisting of micro flakes and micro rods was developed. The optimal monomer-to-oxidant ratio to maintain the pH of the reaction medium above 3.5 was established using low concentrations of aniline (50 mM) and APS (5 mM), with ethanol as the solvent to reduce agglomeration. The synthesized microstructures of sulfonated aniline oligomers were characterized to determine their structure and properties. The mechanisms of the micro sheet formation and the pattern of assembly of the microstructures are also discussed. Furthermore, this research provides a greater understanding of the molecular arrangements of sulfonated aniline oligomers, which can be used for different applications. 

## 2. Results and Discussion

### 2.1. Formation of Sulfonated Aniline Oligomers (SAO)

**Figure 1:**
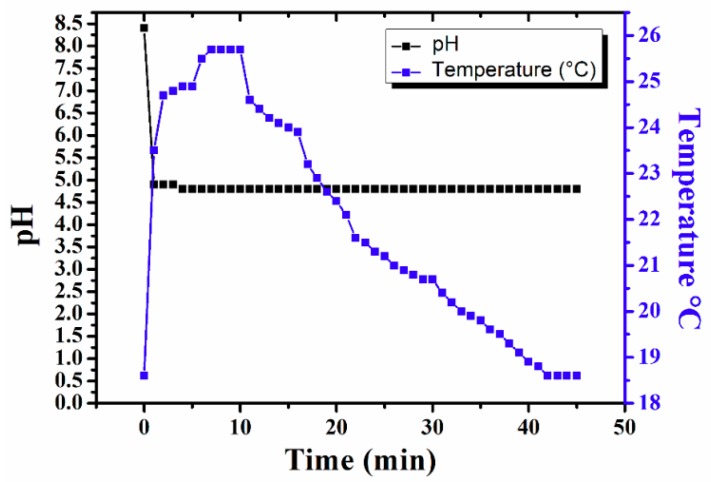
Change in temperature and pH against time over the course of the oxidation of aniline (50 mM) initiated by ammonium persulfate (APS; 5 mM) in 0.13 M HCl solution.

To understand influence of temperature and pH conditions on the synthetic process, the pH- and time-dependence graph plotted against the temperature for the first 45 min is presented in Figure 1. The figure shows a constant pH of 4.8 after 4 min of reaction time, providing ideal pH conditions for oligomer formation. The pH of the aniline dispersed in ethanol immediately dropped from 8.4 to 4.8 after the addition of APS in 0.13 M HCl solution. The temperature of the reaction medium simultaneously increased from 18.6 to 25.7 °C, showing that an exothermic reaction between neutral aniline molecules and APS occurred to produce aniline oligomers [13]. The continuous drop in temperature after 10 min shows that the reaction took place only for a short period of time. Since a low concentration of APS was used, it was completely consumed by the excess neutral aniline present in the reaction medium within a short period. The pH of the reaction medium greater than 3.5, along with the evidence of the exothermic reaction, showed that the reaction proceeded via oligomer formation.

**Figure 2:**
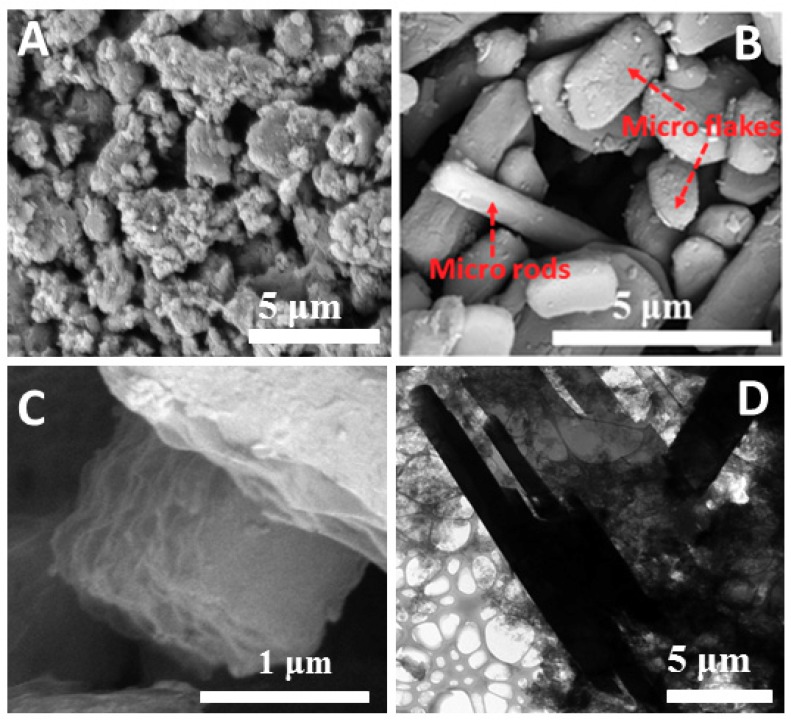
SEM images of the microstructures of the aniline oligomers formed in the presence of reaction media: (**A**) water and (**B**) ethanol. TEM images of (**C**) micro flakes and (**D**) micro rods formed in the presence of ethanol.

The SEM (FEI, Hillsboro, OR, USA) images of the aniline oligomeric structures in the presence of water (Figure 2A) and ethanol (Figure 2B) presented in Figure 2 showed shapeless agglomerates for the polymerization conducted in water, and well-dispersed microstructures, consisting of micro flakes and micro rods, for the reaction performed in ethanol. The TEM images (Figure 2C) show that the micro flakes were formed by the parallel alignment of several individual micro sheets. Figure 2D suggests that the micro sheets were rolled up to form the micro rods. The shapeless aggregates formed in water were similar to the aggregates previously reported by Konyushenko et al., which were synthesized using 200 mM aniline and 25 mM APS in 0.40 M acetic acid medium [13]. However, the microstructures formed in ethanol showed significantly different morphology to the previously reported nanoflakes synthesized by Zujovic et al. [27] in the presence of 24 mM aniline and APS in 4 mM HCl solution.

Previous studies suggest that methanol and ethanol can significantly interfere with aniline to stabilize the synthesized products and suppress agglomeration by forming H-bonds with PANI molecules or wrapping around PANI particles [12,29,30]. A comparison of structures formed using different reaction conditions is shown in Table 1. The advantage of the present conditions is through the adjustment of aniline and APS concentrations where the desired SAO microstructures can be produced with ethanol at a fixed pH.

**Figure 3:**
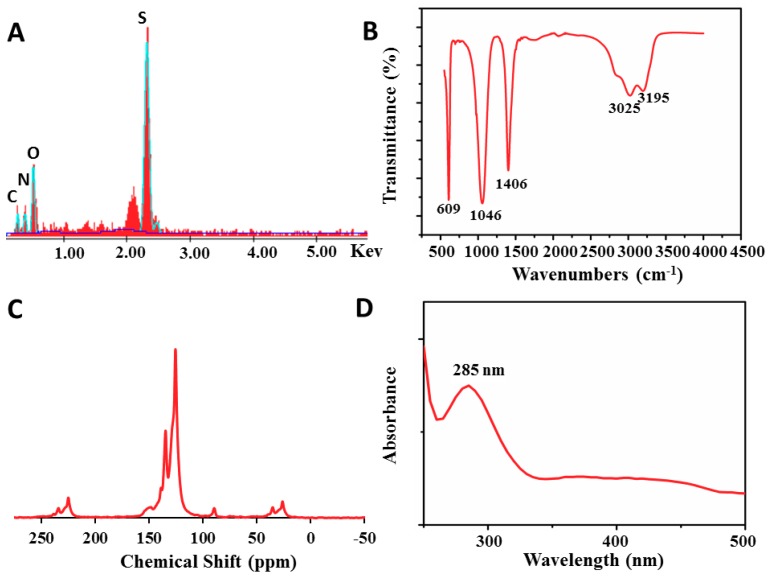
(**A**) energy-dispersive X-ray (EDX), (**B**) Fourier-transform infrared (FTIR), (**C**) ^13^C solid-state NMR, and (**D**) ultraviolet–visible (UV–Vis) spectra of sulfonated polyaniline (PANI).

To investigate and confirm the presence of sulfonate groups in the synthesized sulfonated aniline microstructures, we conducted energy-dispersive X-ray (EDX, Oxford Instruments, Concord, MA, USA) spectroscopy elementary analysis (Figure 3A) of the microstructures, which revealed the presence of 21.97, 18.51, 24.14, and 35.37 wt % of carbon, nitrogen, oxygen, and sulfur, respectively. The high wt % of oxygen and sulfur present in the EDX analysis confirmed that the aniline oligomers were sulfonated. To further analyze the presence of sulfonated groups in the aniline oligomers, we performed Fourier-transform infrared (FTIR) spectroscopy analysis on our products. The FTIR spectrum (Perkin Elmer, Waltham, MA, USA) of aniline oligomers (Figure 3B) shows intense peaks at 3025, 3195, 1414, 1046, and 609 cm^−1^. The peaks at 3025 and 3195 cm^−1^ were ascribed to the C–H aromatic stretching vibration [31] and to hydrogen bonded N–H stretching of aniline oligomers, respectively [25]. The peak at 3195 cm^−1^ could also arise from the –OH groups from –SO_3_H [32]. The band at 1414 cm^−1^ was ascribed to the totally symmetrical stretching of phenazine heterocyclic rings [12,33]. The band at 1046 cm^−1^ is the characteristic peak of the S=O stretching vibration mode in sulfonic groups linked by covalent bonds to the benzene ring [25,34,35]. The peak at 609 cm^−1^ corresponds to the vibration modes of the C–S bond [34,35,36]. These intense peaks observed at 1414, 1046, and 609 cm^−1^ confirmed that the micro sheets consisted of orderly arranged sulfonated phenazine rings. 

Solid-state ^13^C NMR (Bruker, Fällanden, Zürich, Switzerland) was conducted to verify the structure of sulfonated PANI (Figure 3C). The acquired spectrum shows peaks at 125.27, 134.42, and 146.00 ppm corresponding to sp^2^ carbons from the aromatic ring [36,37,38]. The peaks at 224.18, 232.44, 34.53, and 25.29 ppm are spinning side bands derived from the large resonances found near 125.27 ppm. The shoulder found at 138.06 ppm can be attributed to the carbon bound to the phenyl –SO_3_H group, and confirms the formation of the sulfonated polyaniline [38,39,40]. The ultraviolet–visible (UV–Vis, UV-1601, Shimadzu Corporation, Japan) spectrum of SAO (Figure 3D) shows a single peak at 285 nm, ascribed to the phenazine moieties formed [41,42] via strong π–π interactions [21,43]. The absence of peaks below 285 nm shows that the oligomers were not conjugated [44]. The low conductivity (2.04 × 10^−8^ Scm^−1^) measured on the SAO also confirmed that the oligomers were not conjugated.

**Figure 4:**
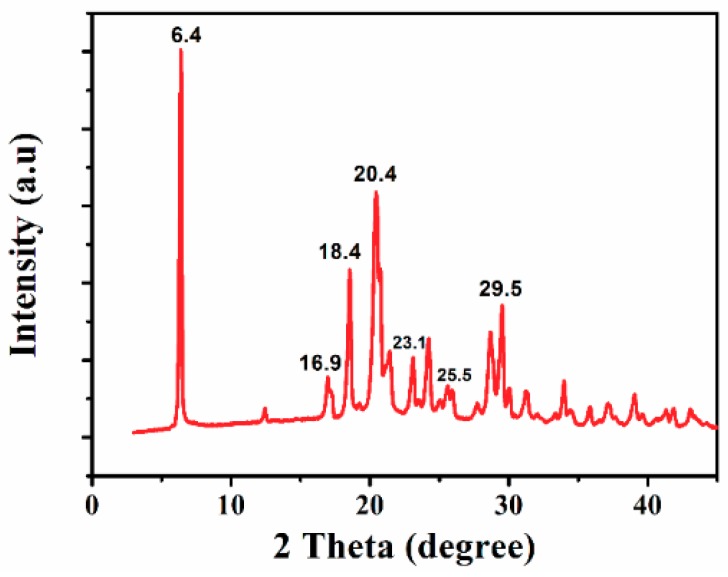
X-ray diffraction (XRD) spectrum of sulfonated PANI oligomers.

The diffraction pattern of SAO was obtained using X-ray diffraction (XRD) analysis (Figure 4). The intense peak at 2θ = 6.4° with a spacing of 13.8 Å is a characteristic peak for periodically aligned polymer chains [26,45,46]. This peak was interpreted differently in several publications, and can arise from the formation of lamella between aniline chains [45], the increase in interlayer distance due to the presence of a dopant [46], or the formation of periodic lamella between PANI chains and sulfate counterions [26]. Therefore, the peak at 2θ = 6.4° was assigned to the periodic lamella between sulfonated oligomeric aniline chains.

The peak at 16.9° (corresponding to a spacing of 5.2 Å) was attributed to the parallel repeat unit of polymer chains [47]. The peaks at 18.4° [26,48] and 20.4° [49] corresponding to d-spacings of 4.7 and 4.3 Å, respectively, were due to the parallel positioning of the polymer chain, whereas the peaks at 25.5° (d-spacing = 3.4 Å) [49] and 29.5° (d-spacing = 3.02 Å) [50] were assigned to the perpendicular arrangement of the polymer chain. The peak at 23.1° could be assigned to the periodicity caused by π–π stacking of oligomers [26]. The XRD pattern confirmed that sulfonated aniline molecules were orderly arranged in a lamella structure. 

### 2.2. Mechanism of Microstructure Formation

**Scheme 1:**
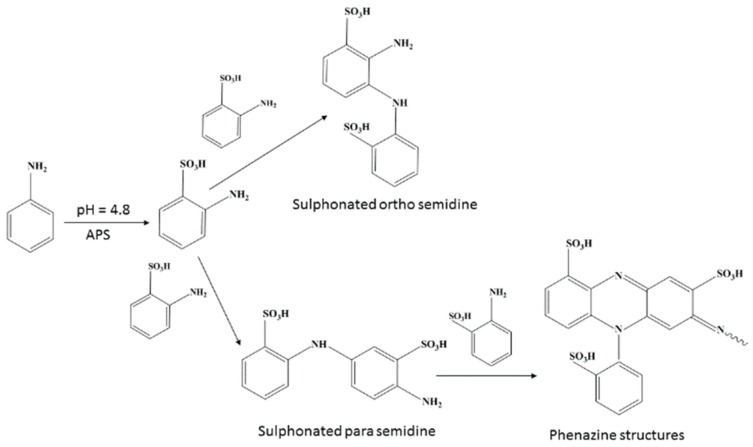
Reaction pathways for the sulfonated PANI oligomers.

Based on the mechanism suggested by Kriz et al. [51] and Stejskal et al. [14] for aniline oligomers, the mechanism for the formation of SAO with phenazine structures is presented in Scheme 1. Initially, both sulfonated *ortho*-semidine and sulfonated *para*-semidine are formed. The sulfonated *para*-semidines then further react with sulfonated aniline molecules to produce planar sulfonated phenazine containing oligomeric structures. 

Previous studies showed that when aniline is oxidized at pH > 3.5 in the presence of neutral aniline, oligomers containing *ortho*- or *para*-substituted monomer units, along with phenazine rings, can be formed [52]. The synthesized product which was characterized by EDX, FTIR, ^13^C NMR, and UV–Vis spectroscopy, as well as XRD, clearly indicated that the microstructures formed were sulfonated and orderly arranged in a phenazine structure. 

**Scheme 2:**
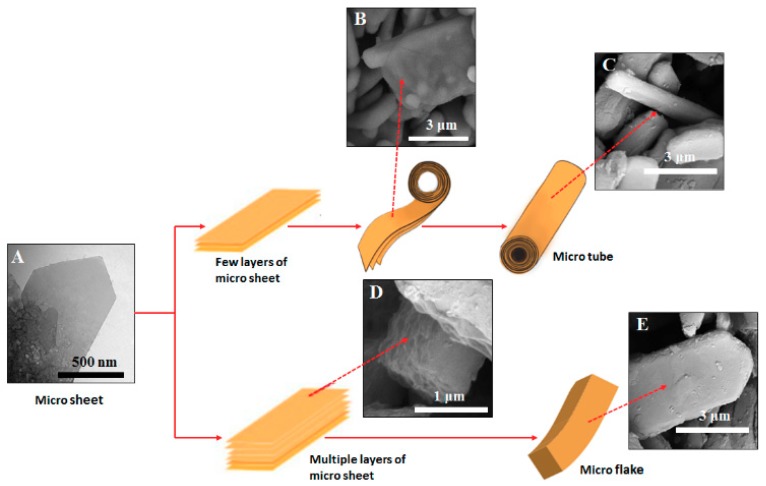
Schematic representation of the formation of micro rods and micro flakes in the product.

Zujovic et al. [27] suggest that these cyclic structures can be interconnected with directed intermolecular hydrogen bonding and π–π interactions to form nanosheets. These bonding interactions align toward an internal ordering and determine the size of the nanosheet. The microstructures formed suggest that the internal ordering of these interconnected cyclic structures via hydrogen bonding and π–π interactions is extended until they form micro sheets. The rapid drop in pH and the immediate increase in temperature in Figure 1 show that rapid intensification of the oligomerization process took place, due to the low amount of APS being fully consumed by the excess of aniline. Viva et al. [53] reported that, at low concentrations of monomers, the formation of head-to-head phenazine structures is favored. As a result, more phenazine structures were formed in the oligomer with a higher degree of uniformity, and aligned toward regular orientation [11]. In particular, aniline trimers containing phenazine moieties are hydrophobic and possess a flat molecular structure, which could stack one-dimensionally via π–π interactions, or roll to reduce their exposed surface energy [14,27]. The SEM conducted on the microstructures did not show any evidence of thin nanorods, but rather showed relatively thick nanorods (width—0.3 μm). Therefore, we assumed that the few layers of micro sheets stacked together by π–π interactions rolled (Scheme 2B) to reduce their surface energy to form micro rods (Scheme 2C). Conversely, the micro sheets aligned as a few layers (Scheme 2D) to reduce the surface energy. The multiple stacking interactions of these micro sheets ultimately lose energy to roll and form nanorods, which eventually settle as micro flakes (Scheme 2E). The synthetic procedure described in this study to obtain well-defined SAO microstructures opens doors to the synthesis of less aggregated, well-dispersed, and well-defined microstructure oligomers with functional groups. 

## 3. Materials and Methods 

### 3.1. Materials 

Aniline (Reagent Plus, 99%) and ammonium persulfate ((NH4)_2_S_2_O_8_, APS) were purchased from Sigma-Aldrich (Saint Louis, MO, USA), and hydrochloric acid (HCl) and ethanol were purchased from Chem Supply (Gillman, Australia).

### 3.2. Methods

#### Preparation of Sulfonated PANI Oligomers

Aniline (5 mL, 5.19 g, 0.05 mol) was dissolved in 40 mL of ethanol, and separately, APS (1.35 g, 0.005 mol) was dissolved in 6 mL of 1 M HCl (aqueous). The solutions were mixed at 5 °C for 30 min using a magnetic stirrer and then continued stirring at room temperature for 16 h. The precipitate was then collected, centrifuged at 4200 rpm, washed with ethanol for 2 h in a Soxhlet extractor, and finally, vacuum-dried overnight at room temperature.

### 3.3. Characterization

The solid-state cross-polarization ^13^C NMR spectrum obtained for sulfonated PANI was acquired on a Bruker 200 Avance spectrometer equipped with a 4.7-T wide-bore superconducting magnet operating at a resonance frequency of 50.33 MHz. The sample was packed into a 7-mm-diameter zirconia rotor with Kel-F end caps and spun at 5 kHz. Chemical shift values were calibrated to the methyl resonance of hexamethylbenzene at 17.36 ppm and a 50-Hz Lorentzian line broadening was applied to the acquired spectrum. The cross-polarization pulse sequence used a 3.2-µs, 195-W, 90° pulse, a contact time of 1 ms, and a recycle delay of 1 s; 50,000 scans were collected. The absolute signal intensity obtained for the sample was corrected for that derived from an empty rotor. Scanning electron microscopy (SEM) images and energy-dispersive X-ray (EDX) spectra were obtained using a Quanta 450 FEI (Hillsboro, OR, USA) at an accelerating voltage of 10 KeV. Samples for SEM were placed on a carbon tape attached to an aluminum stud and coated with platinum. For EDX, three readings were recorded and the average was recorded. X-ray diffraction (XRD) was performed at 40 kV and 15 mA in the range of 2θ = 3–50° at a speed of 10°/min using a Miniflex 600, Rigaku, Japan. Fourier-transform infrared (FTIR) spectroscopy was conducted using a Spectrum 100 (Perkin Elmer, Waltham, MA, USA) using transmission mode. For the UV–Vis analysis of the sample, the sample was dissolved in ethanol and the spectrum was recorded on a Shimadzu UV-1700 spectrometer, Japan. The pH measurements were conducted using a Mettler Toledo pH meter (Mettler-Toledo, Columbus, OH, USA). The pH meter was calibrated using standard buffer solutions before readings were obtained. The electrical conductivity of the SAO was conducted using the following equation:

(1) $$\sigma =\frac{l}{RA},$$

where σ, *l*, *R*, and *A* are the conductivity (S/m), length (m), electrical resistance (Ω), and cross-sectional area (m^2^), respectively. SAOs for the measurement of electrical conductivity were prepared by pressing (50 PSI) them into a 10-mm-diameter cylinder of 1-mm thickness. Conductivity was measured by placing SAOs in between two gold-plated glass electrodes and connected to a two-probe digital multimeter (Fluke −87 V) to measure the resistance. The conductivity was calculated using the resistance, length, and area in Equation (1). 

## 4. Conclusions

This study demonstrates a synthetic procedure to synthesize sulfonated aniline oligomers with well-defined microstructures comprising micro sheets and micro rods in the presence of ethanol. A comparison of the synthesis conducted in the presence of water showed the formation of shapeless aggregates, confirming that ethanol significantly reduces the agglomeration of microstructures. The ideal pH conditions (pH 4.8) were confirmed under which neutral aniline could be present in the reaction medium to perform oligomer formation, and they were achieved using a 10:1 aniline/APS molar ratio. The excess aniline present in the mixture rapidly consumed the APS and formed inter-connected phenazine structures via hydrogen bonding and π–π interactions to form microstructures. The mechanism of the formation of the microstructures was revealed to go through several stages forming two-dimensional planar sheets before their transformation into tubular and layered flake structures. The process to synthesize these sulfonated aniline oligomeric microstructures with a phenazine structure is scalable and controllable toward different morphologies that could be used for a wide range of industrial and medical applications. Phenazine structures and their derivatives are widely explored as antitumor, antibacterial, anti-fungal, and anti-leprosy agents and these products can be adapted for these applications.

## Figures and Tables

**Table 1:** Comparison of the microstructures formed using different reaction conditions.

Aniline Concentration (mM)	APS Concentration (mM)	Products Formed	Medium	Structures Formed			Reference
				Aggregates	Flakes (μm)	Rods/Tubes (μm)	
0.20	0.25	PANI	Water	Shapeless aggregates	-	-	[12]
0.20	0.25	PANI	Methanol	-	-	Tube OD = 0.2	[12]
200	25	AO	Water	Shapeless aggregates	-	-	[13]
24	24	AO	Water	-	L = 1.5 W = 1.0 T = 0.01	-	[27]
50	5	SAO	Water	Shapeless aggregates	-	-	This study
50	5	SAO	Ethanol	-	L = 3.0 W = 2.0 T = 0.4	Rod L = 5.0	This study

APS = ammonium persulfate, PANI = polyaniline, SAO = sulfonated aniline oligomers, L = length, W = width, T = thickness, AO = aniline oligomers, OD = outer diameter.
